# Subthalamic hGAD65 Gene Therapy and Striatum TH Gene Transfer in a Parkinson’s Disease Rat Model

**DOI:** 10.1155/2013/263287

**Published:** 2013-04-29

**Authors:** Deyu Zheng, Xiaohua Jiang, Junpeng Zhao, Deyi Duan, Huanying Zhao, Qunyuan Xu

**Affiliations:** ^1^Beijing Institute for Neuroscience, Beijing Center for Neural Regeneration & Repair, Key Laboratory for Neurodegenerative Disease of the Ministry of Education, Capital Medical University, Beijing 100069, China; ^2^Department of Anatomy, Basic College, Liaoning Medical University, Jinzhou, Liaoning 121001, China; ^3^Medical Center for Experiment and Testing, Capital Medical University, Beijing 100069, China

## Abstract

The aim of the present study is to detect a combination method to utilize gene therapy for the treatment of Parkinson’s disease (PD). Here, a PD rat model is used for the *in vivo* gene therapy of a recombinant adeno-associated virus (AAV2) containing a human glutamic acid decarboxylase 65 (rAAV2-hGAD65) gene delivered to the subthalamic nucleus (STN). This is combined with the *ex vivo* gene delivery of tyrosine hydroxylase (TH) by fibroblasts injected into the striatum. After the treatment, the rotation behavior was improved with the greatest efficacy in the combination group. The results of immunohistochemistry showed that hGAD65 gene delivery by AAV2 successfully led to phenotypic changes of neurons in STN. And the levels of glutamic acid and GABA in the internal segment of the globus pallidus (GPi) and substantia nigra pars reticulata (SNr) were obviously lower than the control groups. However, hGAD65 gene transfer did not effectively protect surviving dopaminergic neurons in the SNc and VTA. This study suggests that subthalamic hGAD65 gene therapy and combined with TH gene therapy can alleviate symptoms of the PD model rats, independent of the protection the DA neurons from death.

## 1. Introduction

The hallmark feature of Parkinson's disease (PD) is degeneration of dopamine neurons in the substantia nigra pars compacta (SNc) and a consequent striatal dopamine deficiency [1–3]. Current clinic treatments for PD mainly focus on alleviating the symptoms with L-3,4-dihydroxyphenylalanine, which increases the synthesis and release of dopamine (DA), particularly during early stages of the disease [4–7]. As the disease progresses, however, pharmacological therapies cannot arrest or reverse neurodegeneration of dopaminergic neurons, and the effectiveness of treatment progressively decreases. Gene therapy techniques may provide an interesting alternative to the treatment of PD. Previous studies have focused on dopamine restoration in the neurotransmitter-depleted and denervated striatum [8, 9] by introducing essential enzymes for dopamine production, such as tyrosine hydroxylase (TH) [10–14], aromatic L-amino acid decarboxylase (AADC) [15, 16], and guanosine triphosphate (GTP) cyclohydrolase I (GTPC-I) [1, 17]. This strategy could potentially increase dopamine levels in the striatum and then improve symptoms in PD rat models.

Actually, the dopamine deficiency in PD leads to a cascade of functional changes in basal ganglia circuitry [2, 3]. The essential pathophysiological characteristic of the PD state is increased neuronal firing activity in the output nuclei of the basal ganglia (globus pallidus pars interna (GPi) and substantia nigra pars reticulata (SNr)) leading to excessive inhibition of thalamocortical and brainstem motor systems [3, 18–20]. This was proposed to arise as a consequence of increased firing in basal ganglia output neurons resulting from reduced inhibition in the “direct” striatal pathway and increased excitation of subthalamic nucleus (STN) consequent to increased activity in striatopallidal GABAergic neurons in the “indirect” pathway [3]. Therefore, it has been proposed that the glutamatergic neurons of the STN can be induced to express GAD and thereby change from an excitatory nucleus to a predominantly inhibitory system that releases GABA at its terminal region in the substantia nigra (SN), leading to suppression of firing activity of these SN neurons. As previously reported, subthalamic GAD gene therapy strategy results in neuroprotection of nigral dopamine neurons and rescue of the parkinsonian behavioral phenotype in a rat model [21]. In that experiment, however, GAD65 gene has been delivered to the STN in rats before 6-OHDA-induced degeneration of dopaminergic neurons. This was not in accordance with the clinical process in PD patients. Therefore, it is not known whether GAD65 gene transfer could have restoration for the remnant dopaminergic neurons against neurodegeneration, improve PD symptoms, and may serve as a potential for treatment of PD.

The present study uses an adeno-associated virus 2 (AVV2) delivery system to introduce hGAD65 into the STN in a rat model of PD. Fibroblasts are utilized as vector cells to transfer the TH gene into the striatum of these rats. Disease symptoms were measured, as well as the number of dopaminergic neurons in SNc and the VTA, and the concentration of glutamic acid and *γ*-aminobutyric acid (GABA). In addition, the neurorestoration of the remnant dopaminergic neurons and potential improvements of symptoms were also monitored. The results demonstrate that the combination rAAV2-hGAD65 and fibroblasts (TH) strategy provide no neurorestoration for remnant dopaminergic neurons in the SNc and VTA in this PD model, but it does alleviate some of the symptoms associated with PD.

## 2. Materials and Methods

### 2.1. Animal

The Sprague-Dawley (SD) rats used in our experiments were from animal center of Capital Medical University. All methods conformed to the Chinese National Health and Medical Research Council published code of practice for the use of animal research and was approved by the Beijing Animal Ethics Committee.

### 2.2. Generation of Recombinant AAV-hGAD65

Human GAD65 was cloned by reverse transcription polymerase chain reaction (RT-PCR) from the cerebral cortices of aborted human embryos (13 weeks of age). Permission was obtained from the pregnant women, and this study was approved by the Ethics Committee of Capital Medical University (Beijing, China). The hGAD65 gene was subcloned into the AAV-2 backbone, which contains a CMV enhancer promoter (Stratagene, USA). Recombinant adeno-associated virus (rAAV-hGAD65) was then packaged and purified [22]. The genomic viral titer was determined using real-time PCR with stocks at a final titer of 1 × 10^11^/mL.

### 2.3. Fibroblasts Cultures

Rat lungs (SD, 1 day old) were removed under aseptic conditions and rinsed in phosphate-buffered saline (PBS) solution (pH 7.4, 1% penicillin and 0.5% streptomycin) for 5 minutes. Pulmonary tissue was separated into 1-2 cm^3^ cubes and digested with 0.25% trypsin for 15 minutes at 37°C. Pulmonary fibroblasts, obtained by centrifugation, were added to culture plates at 3 × 10^6^ cells/mL with Dulbecco's modified essential medium (DMEN, containing 10% fetal bovine serum). Culture medium was replaced every two days, and cells were plated at 70–80% confluency.

### 2.4. Expression of rAAV-hGAD65 *In Vitro*

Fibroblasts were plated at 3 × 10^4^ cells/well into a 24-well plate along with glass coverslips. After 24 hours, 2.0 × 10^8^ of rAAV2-hGAD65 was added to each well in triplicate. After 48 hours, each well was washed with 200 *μ*L artificial CSF (144 mM NaCl, 4 mM KCl, 1 mM MgCl_2_, 5 mM glucose, 1.5 mM CaCl_2_, and pH 7.4). Then, artificial CSF was added to the 24-well plates (100 *μ*L/well) and incubated for 10 minutes prior to collection and analysis of GABA by HPLC [23, 24]. Subsequently, 4% paraformaldehyde (4°C; Sigma, USA) was added to each well (300 *μ*L/well) for 15 minutes, and glass coverslips were rinsed three times with PBS solution (1% triton X-100, pH 7.4) for 5 minutes each. The coverslips were stored at 4°C for immunohistochemistry.

### 2.5. Expression of rAd-TH *In Vitro*

The fibroblasts were plated at 3 × 10^4^ cells/well onto a 24-well plate and the glass slices were put into the 24-well plate. Twenty-four hours later, the fibroblasts were transfected by rAd-TH or rAd vector (reserved by our department) based on the protocol of the transfect kit. After rinsed for 2 times by PBS (pH = 7.2), 2.0 × 10^5^ rAd-TH particles in 200 *μ*L DMEM (contain 10% FBS) were added to the 24-well plates, then the fibroblasts were continually cultured in incubator for 12 hours. Fibroblasts were continuously cultured in incubator after the medium was changed by 10% FBS DMEM. After 48 hours each well was washed with 200 *μ*L DMEM media. Artificial CSF was applied to 24-well plates (100 *μ*L/well) and incubated for 10 minutes before being collected and analyzed. The concentration of dopamine was analyzed by HPLC. Then 4% paraformaldehyde (4°C) was added into each well (300 *μ*L/well) for 15 minutes. The glass slices were rinsed for 5 minutes, 3 times, with PBS solution (1% triton X-100, pH 7.4). Then the slices were stored at 4°C for immunohistochemistry to detect the efficiency of transfection.

### 2.6. Establishment of PD Rat Models

A total of 4 *μ*L 6-hydroxydopamine (6-OHDA) (0.3% 6-OHDA, 0.2% vitamin C, 0.9% physiological saline) was injected into the left SNc and the medial forebrain bundle (MFB) of 190–210 g of the male SD rats using the following stereotaxic coordinates: SNc: −4.4 ± 0.1 AP (from bregma), +1.1 ML, and 7.9 DV, with the incisor bar placed at −2.4 mm below the horizontal zero, MFB: −4.0 ± 0.1 AP (from bregma), +0.8 ML, and 8.1 DV, with the incisor bar placed at +3.4 mm above the horizontal zero. The perfusion rate was 0.8 *μ*L/min, and the needle was left in for an additional 15 minutes. All surgeries were performed under chloral hydrate (36 mg/kg, i.p.) anesthesia.

Apomorphine-induced rotational behaviors were measured using automated rotameter bowls, with tethers attached to the skin of the nuchal region. Apomorphine (0.1 mg/kg, i.p.) was administered at 2 weeks after MFB and SNc lesioned [11, 25, 26]. Rotational behavior was quantified every minute for 40 minutes after allowing the rats to habituate for 5 minutes. Rats that exhibited at least seven full cycles per minute, contralateral to the 6-OHDA-lesioned side, were selected for subsequent experiments.

### 2.7. Injection of rAAV2-hGAD65 and Fibroblasts (TH) into the Rats

Rats were randomly administered combination (rAAV2-hGAD65 (intraSTN injection) and fibroblasts (TH) (intrastriatal injection)) (*n* = 6), rAAV2-hGAD65 (intraSTN injection, *n* = 6), fibroblasts (TH) (intrastriatal injection, *n* = 6), fibroblasts (intrastriatal injection, *n* = 6), rAAV2 vector (intraSTN injection, *n* = 6), or PBS (intraSTN injection), respectively. rAAV2-hGAD65 (4 *μ*L, approximately 4 × 10^8^ genomic particles) plus 1 *μ*L 20% mannitol was unilaterally injected into the STN of 305–385 g of the male SD rats using the following stereotaxic coordinates: −3.8 ± 0.1 AP (from bregma), +2.5 ± 0.1 ML, and 7.3 ± 0.1 DV, with the incisor bar placed at −2.4 mm below the horizontal zero. rAAV2-hGAD65 particles were injected at a rate of 0.3 *μ*L/min, and the needle was left in for an additional 15 minutes. All surgeries were performed under chloral hydrate (36 mg/kg, i.p.) anesthesia. The injection methods of rAAV2 vector and PBS were the same as rAAV2-hGAD65.

Fibroblasts (TH) (fibroblasts transfected by rAd-TH, 10 *μ*L, about 1 × 10^6^ cells) were injected unilaterally into the striatum of 305–385 g, male SD rats using the following stereotaxic coordinates: +0.2 AP (from bregma), +3.0 ± 0.1 ML, and 6.0, 5.5, 5.0, and 4.0 DV (2.5 *μ*L/every point), with the incisor bar placed at −2.4 mm below the horizontal zero. Fibroblasts were infused at the rate of 0.3 *μ*L/min, and the needle was left in for an additional 5 minutes at every point. All surgeries were performed under chloral hydrate (36 mg/kg, i.p.) anesthesia. The injection method of fibroblasts was the same as fibroblasts (TH).

### 2.8. Behavioral Analysis

Apomorphine-induced rotational behaviors were measured using automated rotameter bowls, with tethers attached to the skin of the nuchal region. Apomorphine (0.1 mg/kg, i.p.) was administered at 2 weeks after rAAV2-hGAD65, or rAAV2 vector and PBS injections [23, 26], every 2 weeks until the rats were sacrificed. Rotational behavior was quantified each minute for 40 minutes, once the rats were habituated for 5 minutes. The total number of rotations per minute for a period of 40 minutes was used for analysis.

### 2.9. Immunocytochemistry

At 4, 8, and 16 weeks after the injection of the rAAV2-hGAD65 and/or fibroblasts (TH), the PD rats were deeply anaesthetized with chloral hydrate and perfused intracardially with 0.9% saline, followed by 4% paraformaldehyde (4°C). The brain was removed and placed into 4% paraformaldehyde solution for about 4 hours and then transferred into 30% sucrose solution for 48 hours. Serial, coronal, 40 *μ*m sections were cut at −20°C using a freezing cryostat (Leica, Germany) through the pallidal, subthalamic, and nigral serial levels. Endogenous peroxidase was inactivated with 1% hydrogen peroxide and 70% methanol. Sections were blocked with 10% goat serum and 1% Triton X-100 and then incubated overnight with primary antibody diluted in PBS containing 10% serum [27]. The following primary antibodies were used: mouse anti-human monoclonal hGAD65 (1 : 750, Sigma) and mouse anti-rat monoclonal TH (1 : 12000, Sigma). The secondary antibody was a biotinylated rabbit anti-mouse (1 : 500, Vector Labs, USA), and positive reactions were detected using the Vectastain Elite ABC and DAB substrate kits (Vector Labs, USA).

### 2.10. Measurement of GABA and Glutamic Acid in the SNr and GPi and DA in the Striatum

At 4, 8, and 16 weeks after the injection of rAAV2-hGAD65 and/or fibroblasts (TH), the PD rats were sacrificed by deep anesthesia with chloral hydrate, followed by intracardial perfusion with 0.9% saline. The striatum, SNr, and GPi tissues were separated from the brains under an anatomical microscope. DA, GABA, and glutamic acid concentrations were measured by HPLC [28].

### 2.11. Statistical Analysis

Data are presented as mean ± standard deviation (SD). The ANOVA was used to analysis the data of behavior improvement. Two-sided paired Student's *t*-test was used to compare the mean values of the other data. Differences were considered significant at *P* < 0.05. All statistical analyze were performed using SPSS (version 11.5; SPSS Inc., Chicago, IL, USA).

## 3. Results

### 3.1. Construction and Function of Recombinant rAAV2-hGAD65 Gene *In Vitro*

**Figure 1:**
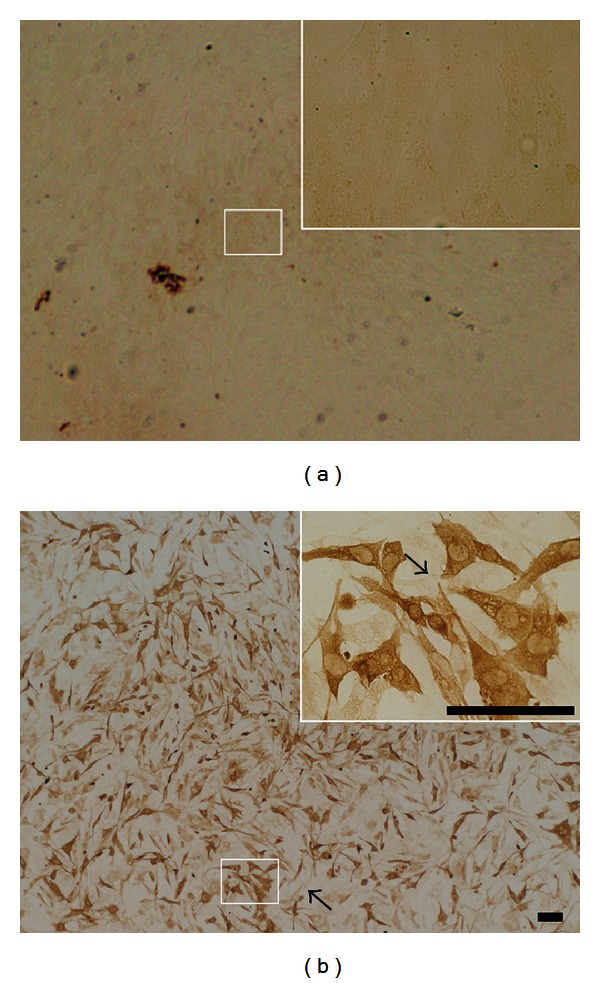
Immunohistochemistry of fibroblasts transfected with rAAV2-hGAD65. (a) It demonstrates the control cells infected by the empty vector. (b) The hGAD65-positive cells were in a brown color in the cytoplasm. Fibroblasts, untransfected by rAAV2-hGAD65, were only shown in their shape (pointed by arrow). The cytoplasm expresses hGAD65, as indicated in the right upper figure. Bar = 100 *μ*m.

After construction of rAAV2-hGAD65, rat fibroblasts were infected with rAAV2-hGAD65 and empty vector, which were confirmed by immunocytochemistry with antibodies specific to hGAD65 (Figure 1). The results of HPLC showed that the concentration of the GABA in the supernatant reached 45.66 ± 6.07 *μ*mol/L.

### 3.2. The Function of TH Gene in Rat Fibroblasts

**Figure 2:**
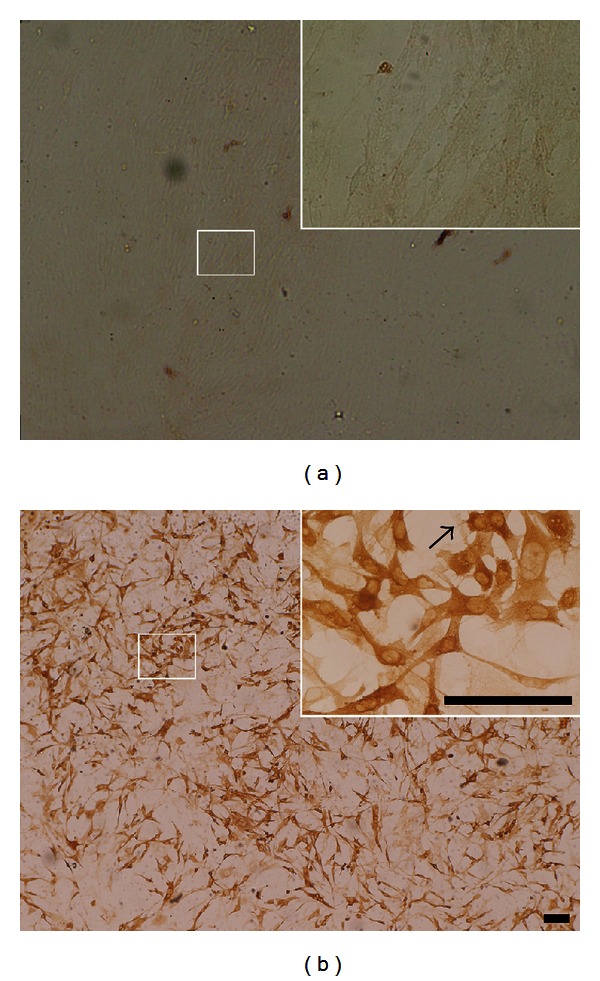
Immunohistochemical analysis of fibroblasts transfected with rAd-TH. (a) It demonstrates the control cells infected by the empty vector. (b) About 85% of the fibroblasts were positive for TH. The untrasfected fibroblasts were only shown in their unclear shape (pointed by arrow). The right upper figure is an enlargement of the main figure. Bar = 100 *μ*m.

Primary rat fibroblasts were cultured from rat lungs and were infected with rAd-TH and empty vector. Expression was confirmed by immunocytochemistry with antibodies specific to TH (Figure 2), which indicated that approximately 85% of the fibroblasts were TH-positive. The untransfected fibroblasts were only showed in their morphologic shape (Figure 2). DA release was quantified by HPLC. The concentration of the DA in the artificial CSF was about 51.34 ± 8.28 nmol/L.

### 3.3. Behavioral Improvement after Injection of Fibroblasts (TH) and/or rAAV2-hGAD65

**Figure 3:**
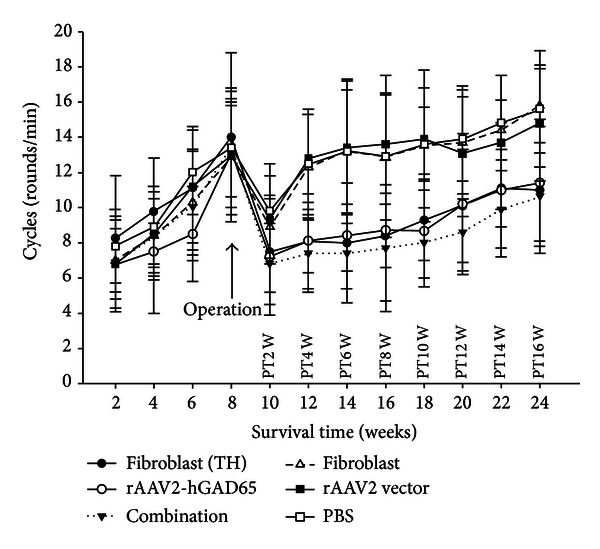
The rotation rate of the subgroup PD rats (arrow indicates the operation, intraSTN injection of rAAV2-hGAD65, rAAV2 vector, or PBS and/or intrastriatal injection of fibroblasts (TH), fibroblast). The cycles of the rats of combination group were a little lower than that of rAAV2-hGAD65, or fibroblasts (TH) groups, but there were no statistical differences between them (*P* > 0.05, *n* = 10). The cycles of the rats of combination, rAAV2-hGAD65, or fibroblasts (TH) groups were all significantly lower than that the control groups (fibroblasts, rAAV2 vector or PBS groups) from 4 to 16 weeks after treatment, respectively (*P* < 0.01, *n* = 10).

Following surgery, the rotation rate was measured after intraperitoneal (i.p.) injection of apomorphine every week until the rats were sacrificed. It showed that the rotation rate significantly decreased (*P* < 0.01) in combination, fibroblasts (TH) or rAAV2-hGAD65 groups (10.78 ± 3.51) from 2 to 16 weeks after treatment compared with that of pretreatment (Figure 3). From 4 to 16 weeks of after treatment, the rotation rates of rAAV2-hGAD65, fibroblasts (TH) groups, respectively, decreased (*P* < 0.05) compared with their control groups (fibroblasts, rAAV2 vector and PBS). In addition, the effect of the combination group was better than that of fibroblasts (TH) or rAAV2-hGAD65 groups but there was no significant difference between them (*P* > 0.05). It also shows that the rotation rates of the combination group had decreased by more than 45% from 4 to 16 weeks after treatment, and their effect continued for 3 months (*P* < 0.01).

### 3.4. Survival of TH-Positive Neurons in the SNc and VTA

**Figure 4:**
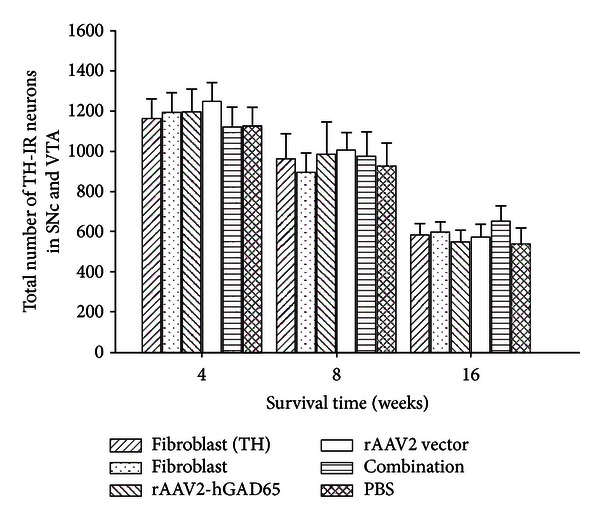
Quantification of tyrosine hydroxylase-positive cells in the SNc and VTA. There was no statistical difference in the number of tyrosine hydroxylase-positive cells in the SNc and VTA between the subgroups of fibroblasts (TH), rAAV2-hGAD65, and combination at 4, 8, and 16 weeks after treatment (*P* > 0.05, *n* = 6). At the same time, there was no statistical difference in the number of the TH-positive neurons in these three groups and the three control groups (fibroblasts, rAAV2 vector, and PBS) (*P* > 0.05, *n* = 6). There was a decreasing tendency of TH-positive neurons of SNc and VTA of all six groups from 4 weeks to 16 weeks after treatment. Fibroblasts (TH), fibroblasts transferred by rAd-TH; fibroblasts, the primary cultured fibroblasts; rAAV2-hGAD65, recombinant adeno-associated virus particles containing human glutamic acid decarboxylase 65; rAAV2 vector, control adeno-associated virus pedicles; combination, rAAV2-hGAD65 + fibroblasts (TH); PBS, inject the PBS into the STN.

**Figure 5:**
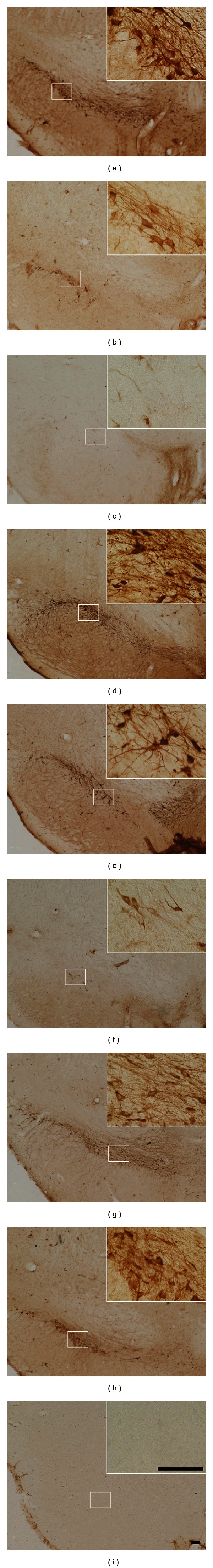
Tyrosine hydroxylase (TH) immunohistochemistry in the SNc and VTA. The numbers of TH-positive cells in the rAAV2-hGAD65 group were similar to that of the fibroblasts- (TH) and combination-treated groups at 4, 8, and 16 weeks after treatment. The lost number of the TH-positive neurons of SNc and VTA was decreased from 4 weeks to 16 weeks after treatment of all these three groups. At first, the cellular morphology was similar to the normal TH-positive neuron in the SNc and VTA (a, b, d, e, g, and h). At week 16, the TH-positive neurons were dramatically reduced, and neurites were shrunken or disrupted. In some model rats, there were no TH-positive neurons in SNc and VTA (c, f, and i). (a, b, and c) fibroblasts (TH) group at 4, 8, and 16 weeks after treatment, respectively; (d, e, and f), rAAV2-hGAD65 group at 4, 8, and 16 weeks after treatment; (g, h, and i), combination group at 4, 8, and 16 weeks after treatment, respectively. Bar = 100 *μ*m.

The rats were sacrificed at 4, 8, and 16 weeks after treatment. Midbrain examination and cell quantification were done using unbiased stereology [29]. The results demonstrated a greater loss of TH-positive neurons in the SNc and VTA (>90% loss) after 4 weeks, compared with the contralateral hemisphere in the all groups (*P* < 0.001). And there was a decreasing tendency of the loss of TH-positive neurons in STN and VTA of all groups from 4 weeks to 16 weeks after treatment (Table 1, Figures 4 and 5). At 4 weeks, the loss ratio of TH-positive neurons (SNc and VTA) of the combination groups was 90.82% and a little more than that of the fibroblasts (TH), or rAAV2-hGAD65 groups, but there was no significant difference between them (*P* > 0.05). It also showed that the loss ratio of TH-positive neurons of the combination was not significantly different compared with other groups at 16 weeks (*P* > 0.05) (Table 1 and Figure 4).

### 3.5. The Concentration of the Dopamine in the Striatum

To test the expression of rAd-TH in the striatum, we analyzed the DA levels of the striatum by HPLC from at 4, 8, and 16 weeks after treatment. The results showed that the DA concentration in the striatum of the rAAV2-hGAD65 group was much lower than that of the rats of the fibroblasts (TH) group or the combination group (*P* < 0.001, Table 2). Though the datum of the combination group rats was higher than that of the fibroblasts (TH) group, there was no statistically significant difference between them (*P* > 0.05, Table 2). The concentration of the DA was almost the same as the control groups (fibroblasts, rAAV2 vector, and PBS) (*P* > 0.05, Table 2).

### 3.6. GABA and Glutamic Acid Levels in the GPi and SNr

**Figure 6:**
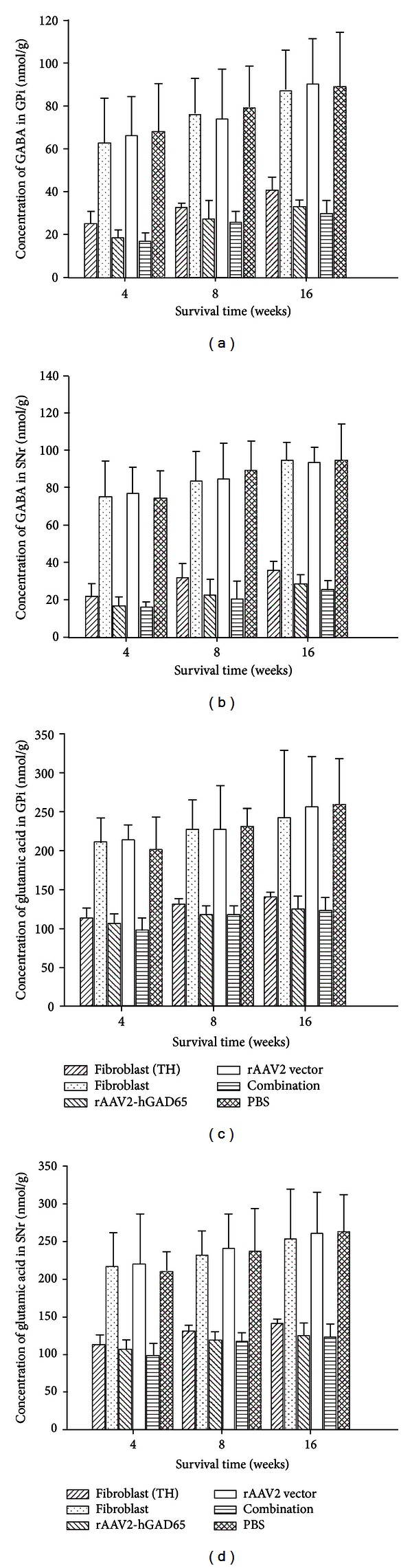
GABA and glutamic acid concentrations in the GPi and SNr tissues. (a and b) The concentrations of GABA in the GPi and SNr tissues of combination group were a little lower than that of rAAV2-hGAD65 and fibroblasts (TH) groups (*P* > 0.05, *n* = 6). But the GABA concentrations in these three groups were all obviously lower than that of the three control groups (fibroblasts, rAAV2 vector, and PBS) at 4, 8, and 16 weeks after treatment (*P* < 0.01, *n* = 6). (c and d) The changing tendency of the concentration of the glutamic acid was similar to the change of the GABA in GPi and SNr at the 4, 8, and 16 weeks after treatment (*P* > 0.05, *n* = 6). Fibroblasts (TH), fibroblasts transferred by rAd-TH; fibroblasts, the primary cultured fibroblasts; rAAV2-hGAD65, recombinant adeno-associated virus particles containing human glutamic acid decarboxylase 65; rAAV2 vector, control adeno-associated virus pedicles; combination, rAAV2-hGAD65 + fibroblasts (TH); PBS, inject the PBS into the STN.

To evaluate rAAV-mediated GAD65 gene transfer, SNr and GPi tissues of the rats in all groups at 4, 8, and 16 weeks after treatment were separated and analyzed for glutamic acid and GABA concentrations using HPLC [30]. The results showed that GABA concentrations in GPi or SNr tissues of the combination group were a little lower than those of fibroblasts- (TH-) or rAAV2-hGAD65-treated groups at 4, 8, and 16 weeks after treatment, respectively, but there was not a significant difference between them (*P* > 0.05, Figures 6(a) and 6(b)). The GABA concentrations in GPi or SNr of the combination-, fibroblasts- (TH-), or rAAV2-hGAD65-treated groups were all much lower than those of three control groups (fibroblasts, rAAV2 vector, or PBS groups), respectively, at 4, 8, or 16 weeks after treatment (*P* < 0.01). In addition, the results displayed that GABA gradually increase from 4 weeks to 16 weeks post-treatment of all groups. The tendency of glutamic acid concentrations was similar to the GABA performance (Figures 6(c) and 6(d)).

### 3.7. Human GAD65 Expression in the STN

**Figure 7:**
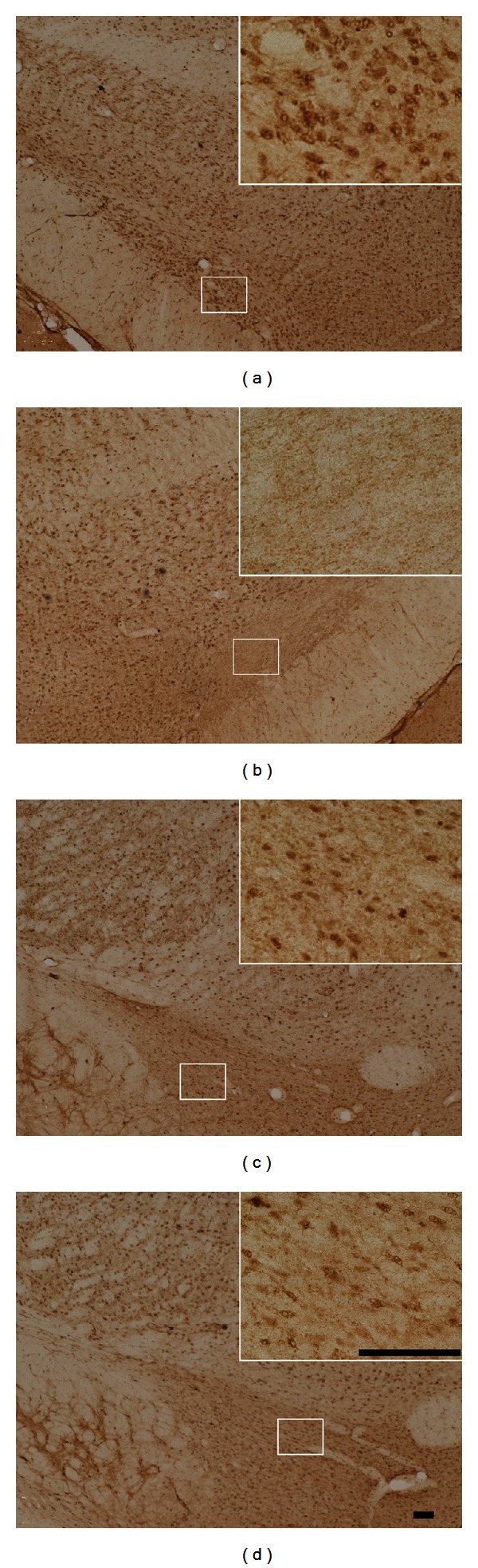
Immunohistochemistry of hGAD65 in the STN (DAB, bar = 100 *μ*m). A large number of hGAD65-positive cells are observed in the left STN (a, c, and d) of rAAV2-hGAD65 groups, but there are no positive cells in either hemispheres of the TH group (b). The number of hGAD65-positive cells decreased according to posttreatment time: 4 weeks (a), 8 weeks (c), and 16 weeks (d).

The results of hGAD65 immunohistochemistry showed that hGAD65-positive cells were observed in the injected side of the rAAV2-hGAD65-treated rats and in the combination-treated rats at 4, 8, and 16 weeks after treatment (Figures 7(a), 7(c), and 7(d)). However, hGAD65 was not expressed in cells on the contralateral hemispheres of all groups, or in either ipsilateral hemispheres of the fibroblasts (TH) group rats (Figure 7(b)). The numbers of hGAD65-positive cells of rAAV2-hGAD65 group (703.2 ± 45.3, 674.5 ± 21.5, and 609.3 ± 26.7) and combination group (721.4 ± 66.7, 667.2 ± 32.9, and 618.5 ± 45.5) were decreased in the time course, but there was no significant difference between them (*P* > 0.05) (Table 3, Figures 7(a), 7(c), and 7(d)). There was a correlation in time course between the number of hGAD65-positive neurons of STN and glutamic acid or GABA levels of the GPi and SNr (Figures 6 and 7, Table 3).

### 3.8. Survival of Fibroblasts Expressing the TH in the Striatum

**Figure 8:**
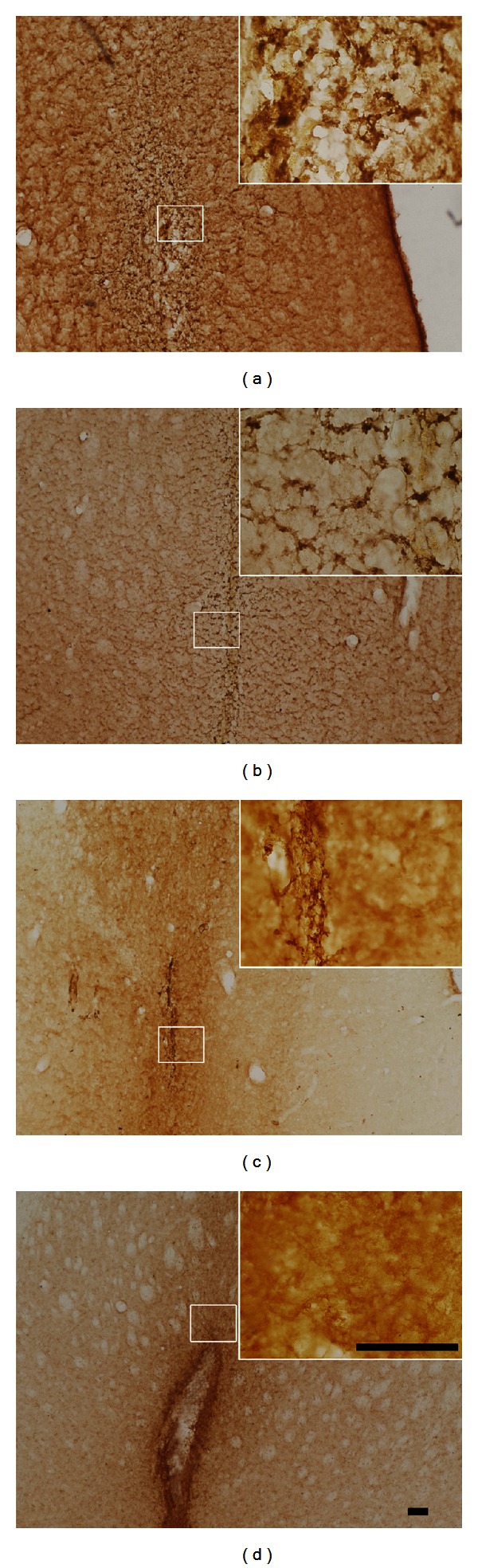
Immunohistochemistry of fibroblasts expressing TH in the striatum (DAB, bar = 100 *μ*m). A large number of TH-positive cells are observed in the left striatum around the pin track in fibroblasts (TH) group, but there are no positive cells in either hemispheres of the rAAV2-hGAD65 group. The number of TH-positive cells decreased according to posttreatment time: 4 weeks (a), 8 weeks (b), and 16 weeks (c). There was no TH-positive cell around the pin track in the left striatum of fibroblast at 4 weeks after treatment (d).

The TH immunoreactive fibroblasts in the striatum were detected by immunohistochemistry. It showed that the TH-immunoreactive fibroblasts in the striatum were time-dependently reduced from 4 weeks to the 16 weeks after treatment. The TH-immunoreactive fibroblasts could be seen in the area surrounding the injection site 4 weeks after the surgery (Figure 8(a)). A few of the TH-immunoreactive fibroblasts emigrated from the pin holes at 8 weeks after treatment (Figure 8(b)), and the fibroblasts could be seen within the pin holes 16 weeks after treatment (Figure 8(c)). In the striatum of control group rats (fibroblasts treated), however, there was no TH-positive cell around the pin track (Figure 8(d)).

## 4. Discussion

GABA is the most widely distributed inhibitory neurotransmitter in the vertebrate brain [31–33]. In addition, the brain contains two isoforms of GABA-synthesizing enzymes, GAD65 and GAD67, which differ in molecular size, amino acid sequence, antigenicity, and cellular and subcellular localizations [34–36]. GAD65 functions were executed more as a neurotransmitter maker than does GAD67 [21, 37–39]. In PD, dopaminergic neurons of SNc are gradually degenerated for some reasons, leading to marked diminution of the dopamine concentration in the striatum, the primary projection region. The major inhibitory output nuclei of the basal ganglia, SNr and GPi, as a result, are overexcited driven by a disinhibited and thereby overactive subthalamic nucleus [19, 20, 40]. This further induces the canonical symptoms of PD, that is, trembling, dyskinesia, rigidity, and so forth.

In the present study the behavioral, immunohistochemical, and neurochemical analyses strongly support the hypothesis that *in vivo* gene delivery of hGAD65 by AAV2 (intraSTN-hGAD65) improves motor symptoms in rat models. hGAD65 gene expression, induced by AAV2, led to phenotypic changes in excitation neurons and inhibitory neurons, *via* enzymatic action from glutamic acid to GABA. These phenotypic changes resulted from inhibition of the neurons of the GPi and SNr for the overactivation of subthalamic nucleus in the PD model rats. This method improved motor symptoms by inhibiting the efferent basal ganglial loop (GPi and SNr). In addition, the excitation patterns of GPi and SNr were decreased.

The present results demonstrate that there is no significant protective function of the surviving dopaminergic neurons in the SNc and VTA from hGAD65 gene expression in the STN. These results are not consistent with previous results [21], which could be due to the experimental stages. In the previous study, hGAD65 gene was injected into the STN, followed by medial forebrain bundle lesion by 6-OHDA. In our study, however, MFB and SNc 6-OHDA lesions were followed by hGAD65 gene transfer. So it could be more similar to the disease progression in Parkinson's patients than in the previous study [21]. In Parkinson's patients or PD model rats, an unknown mechanism causes dopaminergic neurons to undergo degeneration and necrosis. As previously reported, once dopaminergic neuronal degeneration or necrosis is underway, the latency period could be longer than 10 years [2, 10, 18, 27]. The results here showed that hGAD65 gene transfer did not effectively protect surviving dopaminergic neurons in the SNc and VTA. In addition, it also demonstrated that although the improvement effect of the combination method on the motor symptoms of the PD model rats did better than hGAD65 gene transfer and TH delivery alone, there is no significant protective function for surviving dopaminergic neurons in the SNc and VTA.

In conclusion, the present study suggests that the methods of hGAD65 gene injection into the STN and the combination of the hGAD65 gene transfer and TH gene therapy into the striatum can improve the phenotype shift of the PD model rats, independently of the protection of the DA neurons from death.

## Figures and Tables

**Table 1:** Quantification of tyrosine hydroxylase-positive neurons in the SNc and VTA (*n=6*).

	Fibroblasts (TH)		rAAV2-hGAD65		Combination		Fibroblasts		rAAV2 vector		PBS	
	Number	Percent of control	Number	Percent of control	Number	Percent of control	Number	Percent of control	Number	Percent of control	Number	Percent of control
4 W	1169.3 ± 99.3	9.52 ± 0.81%	1198.5 ± 109.7	9.76 ± 0.89%	1127.5 ± 0.54	9.18 ± 0.81%	1201.3 ± 96.4	9.68 ± 0.94%	1251.4 ± 91.3	9.79 ± 2.01%	1129.4 ± 89.3	9.05 ± 1.92%
8 W	966.0 ± 121.1	8.24 ± 1.03%	988.3 ± 157.8	8.43 ± 1.35%	978.8 ± 0.41	8.35 ± 1.01%	898.5 ± 102.4	8.17 ± 1.20%	1013.2 ± 93.2	8.74 ± 1.36%	928.2 ± 112.4	8.23 ± 1.67%
16 W	587.5 ± 59.6	4.78 ± 0.71%	551.5 ± 53.5	4.49 ± 0.43%	650.8 ± 0.41	5.31 ± 0.77%	602.3 ± 47.9	5.24 ± 0.84%	579.2 ± 65.3	4.86 ± 0.93%	542.4 ± 82.2	4.37 ± 0.89%

The number of TH-positive neurons was in a decreasing tendency in all groups from 4 weeks to 16 weeks after treatment. There was no significant difference of number of TH-positive neurons compared to each other at 4, 8, and 16 weeks after treatment, respectively. Fibroblasts (TH), fibroblasts transferred by rAd-TH; rAAV2-hGAD65, recombinant AAV2 particles, containing human glutamic acid decarboxylase 2 gene; combination, rAAV2-hGAD65 + fibroblasts (TH), fibroblasts, primary cultured fibroblasts, rAAV2 vector, recombinant AAV2 particles, PBS, pH = 7.2, 0.1 M.

**Table 2:** Concentration of DA in the striatum (nmol/L, *n* = 6).

	Fibroblasts (TH)		rAAV-hGAD65		Combination		Fibroblasts		rAAV2 vector		PBS	
	Concentration	Percent of control	Concentration	Percent of control	Concentration	Percent of control	Concentration	Percent of control	Concentration	Percent of control	Concentration	Percent of control
4 W	5.58 ± 0.65**	10.54 ± 1.23%	1.22 ± 0.23^∗∗##^	2.30 ± 0.43%	6.41 ± 0.54^##^	12.10 ± 0.97%	1.24 ± 0.25^∗∗##^	2.32 ± 0.27%	1.30 ± 0.26^∗∗##^	2.41 ± 0.51%	1.31 ± 0.29^∗∗##^	2.46 ± 0.71%
8 W	4.33 ± 0.74**	8.17 ± 1.41%	1.04 ± 0.16^∗∗##^	1.97 ± 0.30%	4.80 ± 0.41^##^	9.07 ± 0.78%	1.03 ± 0.31^∗∗##^	1.96 ± 0.34%	1.09 ± 0.26^∗∗##^	2.02 ± 0.47%	1.10 ± 0.21^∗∗##^	2.11 ± 0.54%
16 W	3.77 ± 0.37**	7.11 ± 0.70%	0.59 ± 0.15^∗∗##^	1.10 ± 0.27%	4.20 ± 0.41^##^	7.93 ± 0.78%	0.61 ± 0.15^∗∗##^	1.11 ± 0.30%	0.65 ± 0.21^∗∗##^	1.13 ± 0.44%	0.67 ± 0.23^∗∗##^	1.16 ± 0.28%

Statistical significance (**, ^##^) in the concentrations of DA in the striatum between the rAAV2-hGAD65-treated group and the TH-treated group or the combination-treated group, at 4, 8, and 16 weeks after treatment (*P* < 0.001, *n* = 6). The DA level of rAAV2-hGAD65 group was similar to that of three control groups (fibroblasts, rAAV2 vector, and PBS groups at 4, 8, and 16 week after treatment, respectively (*P* > 0.05, *n* = 6). Fibroblasts (TH), fibroblasts transferred by rAd-TH; rAAV2-hGAD65, recombinant adeno-associated virus particles, containing human glutamic acid decarboxylase 2 gene; combination, rAAV2-hGAD65 + fibroblasts (TH), fibroblasts, primary cultrued fibroblasts, rAAV2 vector, recombinant AAV2 particles, PBS, pH = 7.2, 0.1 M.

**Table 3:** The number of the hGAD65-positive neurons in STN.

	rAAV2-hGAD65	Combination
4 W	413.2 ± 45.3	421.4 ± 66.7
8 W	391.5 ± 21.5	397.2 ± 32.9
16 W	353.3 ± 26.7	360.5 ± 45.5

There were no significant differences between rAAV2-hGAD65 group and combination group at 4, 8, and 16 weeks after treatment, respectively (*P* > 0.05, *n* = 6).
